# Evidence linking rapid Arctic warming to mid-latitude weather patterns

**DOI:** 10.1098/rsta.2014.0170

**Published:** 2015-07-13

**Authors:** Jennifer Francis, Natasa Skific

**Affiliations:** Department of Marine and Coastal Sciences, Rutgers University, New Brunswick, NJ, USA

**Keywords:** Arctic, extreme weather, jet stream

## Abstract

The effects of rapid Arctic warming and ice loss on weather patterns in the Northern Hemisphere is a topic of active research, lively scientific debate and high societal impact. The emergence of Arctic amplification—the enhanced sensitivity of high-latitude temperature to global warming—in only the last 10–20 years presents a challenge to identifying statistically robust atmospheric responses using observations. Several recent studies have proposed and demonstrated new mechanisms by which the changing Arctic may be affecting weather patterns in mid-latitudes, and these linkages differ fundamentally from tropics/jet-stream interactions through the transfer of wave energy. In this study, new metrics and evidence are presented that suggest disproportionate Arctic warming—and resulting weakening of the poleward temperature gradient—is causing the Northern Hemisphere circulation to assume a more meridional character (i.e. wavier), although not uniformly in space or by season, and that highly amplified jet-stream patterns are occurring more frequently. Further analysis based on self-organizing maps supports this finding. These changes in circulation are expected to lead to persistent weather patterns that are known to cause extreme weather events. As emissions of greenhouse gases continue unabated, therefore, the continued amplification of Arctic warming should favour an increased occurrence of extreme events caused by prolonged weather conditions.

## 1. Introduction

**Figure 1.**
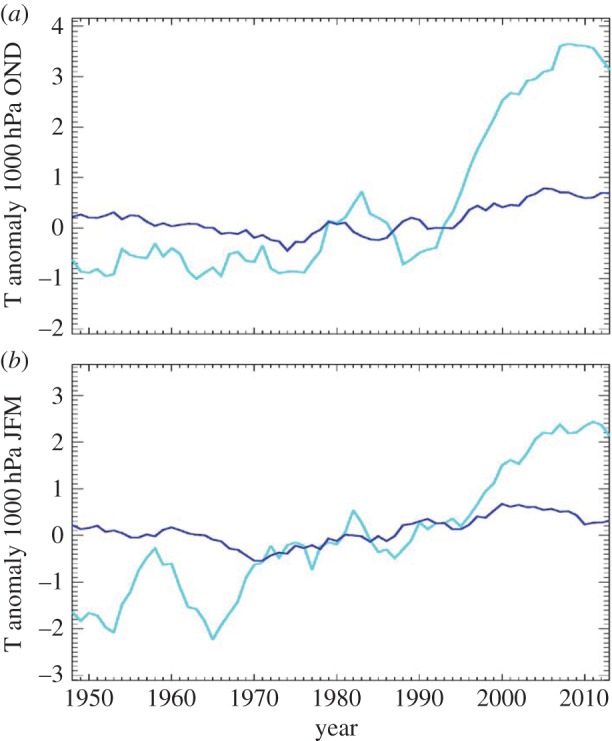
Five-year running means of near-surface air temperature anomalies (C, relative to 1970–1999) during autumn (Oct.–Dec., *a*) and winter (Jan.–Mar., *b*) for the Arctic (70° N to 90° N, cyan) and for the Northern Hemisphere mid-latitudes (30° N to 60° N, blue). Data were obtained from the NCEP Reanalysis, NOAA/ESRL Physical Sciences Division, Boulder, CO, http://www.esrl.noaa.gov/psd/.

A variety of positive feedbacks—processes that amplify an original change—cause the Arctic to be more sensitive to global temperature change than anywhere else on the Earth (e.g. [1,2]). This heightened sensitivity is known as Arctic amplification (AA). Consequently, the Arctic's lower tropospheric air temperature has continued to rise at least twice as fast as in the Northern Hemisphere's mid-latitudes during recent decades (figure 1). AA is largest in autumn and winter, with weaker signals emerging in spring and summer ([3]; hereafter, FV15). Recent studies suggest that the rapidly warming Arctic is associated with an increase in extreme weather events, such as cold spells [4,5] and heat waves [6,7] in Northern Hemisphere continents, as well as wet summers and flooding in Eurasia [6,8]. Identifying the mechanism(s) underlying the linkage is a focus of active research, including an assessment of the relative roles of anthropogenic forcing versus random natural variations in causing these events.

Several mechanisms have been proposed that explain how AA may influence the large-scale circulation of the Northern Hemisphere and, in particular, the polar jet stream. One hypothesis connects AA to more persistent mid-latitude weather patterns through its effects on the configuration of the jet stream. AA has reduced the Arctic/mid-latitude temperature contrast in recent decades, particularly during autumn and winter ([9]; FV15). Because this temperature gradient is a fundamental driver of the jet stream's westerly (zonal) wind speed, the weaker gradient leads to slower zonal jet-stream winds [10–12]. A slower jet stream tends to take a more meandering (meridional) path as it encircles the Northern Hemisphere [13,14]. Large north–south jet-stream waves in a highly meandering flow tend to travel eastward more slowly. These waves create the high- and low-pressure systems at the surface, so their slower eastward progression increases the likelihood of persistent weather patterns that can cause a variety of extreme events [15].

Several recent studies have identified a mechanism linking ice loss in the Barents/Kara Seas (BK) area of the Arctic Ocean during autumn with anomalously cold winters in central Asia [16–18]. This mechanism is summarized in the schematic in figure 2 from Cohen *et al*. [16]. When ice coverage is low in BK, large heat fluxes from the insolation-warmed ocean transfer energy into the overlying atmosphere, which creates a geopotential height anomaly in atmospheric layers above. This contributes to anomalous upper-level ridging (northward extension of the jet stream) in the region, which tends to strengthen surface high-pressure downstream. The clockwise circulation around the high transports cold air from the Arctic into central Asia, which leads to cold spells as well as distending the trough (southward dip) of the jet stream over the area. The result of the enhanced ridge/trough couplet is an increase in wave energy, which is then transferred into the stratosphere, where it can disrupt the polar vortex and lead to further meandering in the jet stream later in winter. The fact that these three independent studies identified the same mechanism using different approaches, data sources and model experiments suggests that the linkage is robust.

**Figure 2.**
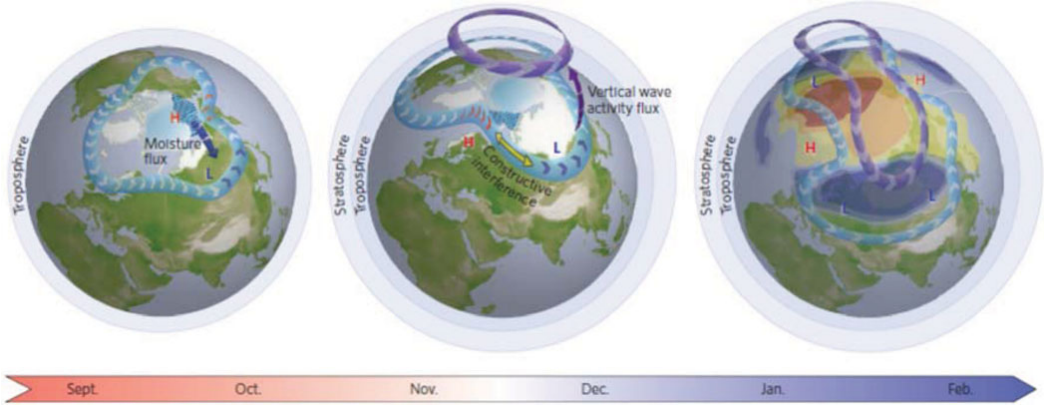
Arctic sea ice along with earlier and more extensive Eurasian snow cover in the autumn may favour the negative phase of the NAO/AO in winter. Snow is shown in white, sea ice in light blue, sea ice melt with blue waves, anomalous high and low geopotential heights with red ‘H’ and blue ‘L’, tropospheric polar jet stream in light blue with arrows, and stratospheric polar vortex in purple with arrows. Middle and right diagrams illustrate cold (warm) temperature anomalies associated with the negative phase of the winter NAO/AO, shown in blue (orange). Adapted from [16].

A primarily summer mechanism proposed and demonstrated by Petoukhov *et al*. [19] and Coumou *et al.* [6] suggests that the weakened poleward gradient owing to AA is conducive to the formation of a split-jet structure during summer months that acts as a waveguide, trapping and amplifying large jet-stream waves. These waves then become nearly stationary, creating persistent weather conditions that have contributed to an increasing number of temperature and precipitation extremes over Northern Hemisphere continents.

In this paper, new research is discussed that provides further evidence that rapid Arctic warming is causing a more meandering jet stream. Most Arctic/jet-stream linkages are distinct from tropical/jet-stream linkages in that they do not directly involve the injection of wave energy into the jet stream, such as that identified by Ding *et al.* [20] and proposed by Palmer [21] to explain the ‘polar vortex’ of winter 2013/2014. This new manifestation of global warming through AA may have substantial societal impact, as more frequent extreme weather events in mid-latitudes will affect billions of people directly through damage to property and infrastructure, and indirectly through agriculture and water supplies. Moreover, the amplified patterns exhibit regional preferences for anomalies in temperature and precipitation (e.g. [15,17]), thus it may be possible to predict which types of extreme events will be more likely to occur in certain areas and, in turn, assist decision-makers in preparing for the future. However, because the atmosphere is inherently chaotic and the signal of AA has emerged only recently, it is a challenge to detect robust changes in the character of the jet stream [22,23] and separate the various influences on its behaviour.

## 2. Evidence linking Arctic amplification to a more meridional upper-level flow

**Figure 3.**
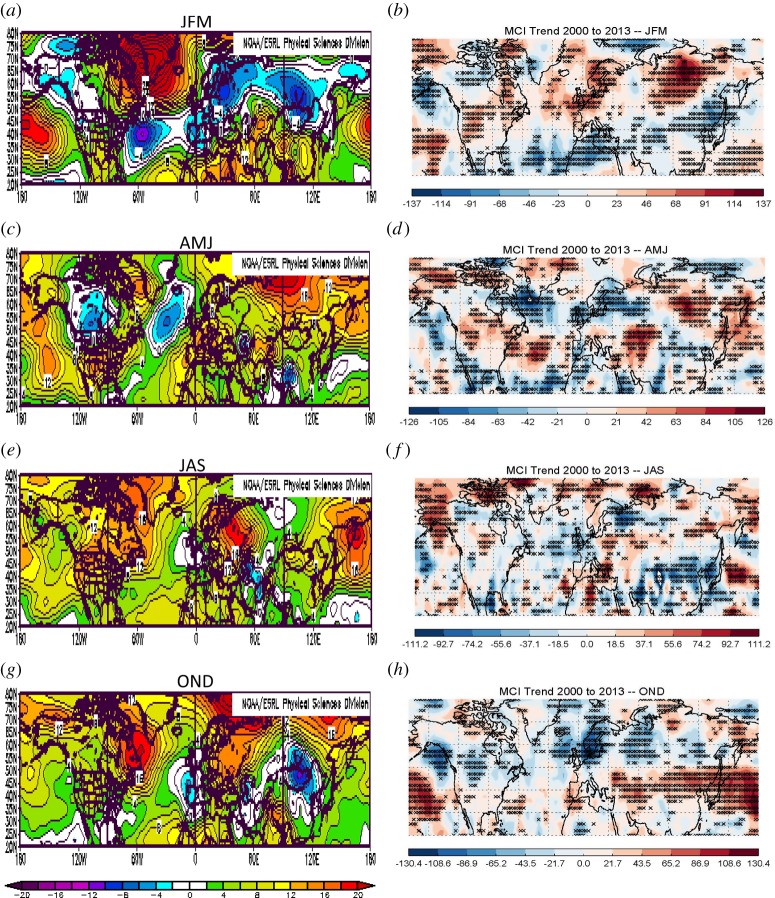
(*a*,*c*,*e*,*g*) Seasonal anomalies in the 1000-to-500 hPa thickness during 2000 to 2013 relative to 1981 to 2010 (m). (*b*,*d*,*f*,*h*) Seasonal trends in |MCI| during 2000 to 2013 (year^−1^×10^5^). Asterisks denote significant trends with a 95% confidence.

High latitudes are warming more rapidly than are mid-latitudes, but the spatial distribution of the temperature changes is not uniform. One measure of the warming over the lower atmosphere is the change in 1000-to-500 hPa atmospheric thickness. Figure 3*a*,*c*,*e*,*g* presents seasonal anomalies in thickness over the Northern Hemisphere during 2000 to 2013 relative to climatology (1981–2010), elucidating changes during the recent period when AA clearly emerged from the noise of natural variability (figure 1). The colours indicate that thickness anomalies are mostly positive, reflecting the general warming of the globe. The autumn (OND) patterns are the most Arctic-Ocean-centric owing to effects of sea-ice loss, while other seasons are highly spatially variable. Changes in the poleward thickness *gradients*, therefore, will also be highly spatially variable, so attempts to analyse hemispheric-mean responses of the circulation may not be especially helpful in understanding mechanisms and processes leading to jet-stream and weather changes. A more localized approach is in order.

Varying poleward gradients, through the thermal wind relationship, will affect the speed of the upper-level zonal winds: in areas where the gradient strengthens (weakens), zonal winds strengthen (weaken), as demonstrated by FV15. These wind changes will also affect the wind *direction*. For example, a reduction in the zonal component of the wind at any particular location will cause, through simple vector geometry, the wind vector to become more meridional. Owing to AA, this effect alone results in an overall increased meridional character to the flow, although the effects will vary greatly regionally and seasonally depending on where the spatial patterns of warming/cooling are focused. A simple measure of the flow character has been developed (FV15), the meridional circulation index (MCI):

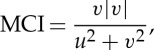
where *u* and *v* are the zonal and meridional components of the wind, respectively. When MCI>0.5, the wind is predominantly southerly, when MCI<−0.5, it is predominantly northerly, and when −0.5<MCI<0.5, the wind is mainly zonal. The absolute value of MCI gives the overall ‘meridionalness’ of the wind, with a value greater than 0.5 indicating predominantly meridional flow. Figure 3*b*,*d*,*f*,*h* presents seasonal trends in |MCI| for 2000 to 2013 over the Northern Hemisphere from 20 to 80° N for winds at the 500 hPa level. Red (blue) areas indicate where winds have become more meridional (zonal), and asterisks denote significant trends at the 95% confidence level (using a *χ*^2^ goodness-of-fit test).

It is clear that a close correspondence exists between changes in thickness anomalies (figure 3*a*,*c*,*e*,*g*) and trends in |MCI|. Increasing |MCI| tends to be located in areas where the poleward thickness gradients have weakened, indicated by positive thickness anomalies located northward or negative anomalies southward of a location. During autumn and winter particularly, the mid-latitudes exhibit predominantly increased |MCI|, which are the seasons when AA has been strongest. These results support the existence of a direct linkage between disproportionate Arctic warming in recent years and a wavier jet-stream configuration, as proposed by Francis & Vavrus ([11]; hereafter, FV12) and Liu *et al.* [12]. Further results using the MCI metric appear in FV15.

## 3. Increased frequency of amplified jet-stream patterns

Highly amplified jet-stream patterns (HAPs) are responsible for many extreme weather events [15]; therefore, an increased frequency of these patterns would be expected to result in an increase in extreme weather events. FV12 hypothesized, and presented evidence showing, that the meridional amplitudes of planetary waves should increase in response to AA. The approach presented here is somewhat different from that in FV12, in that the *frequency* of amplified jet-stream configurations is analysed rather than changes in the amplitude of the waves.

**Figure 4.**
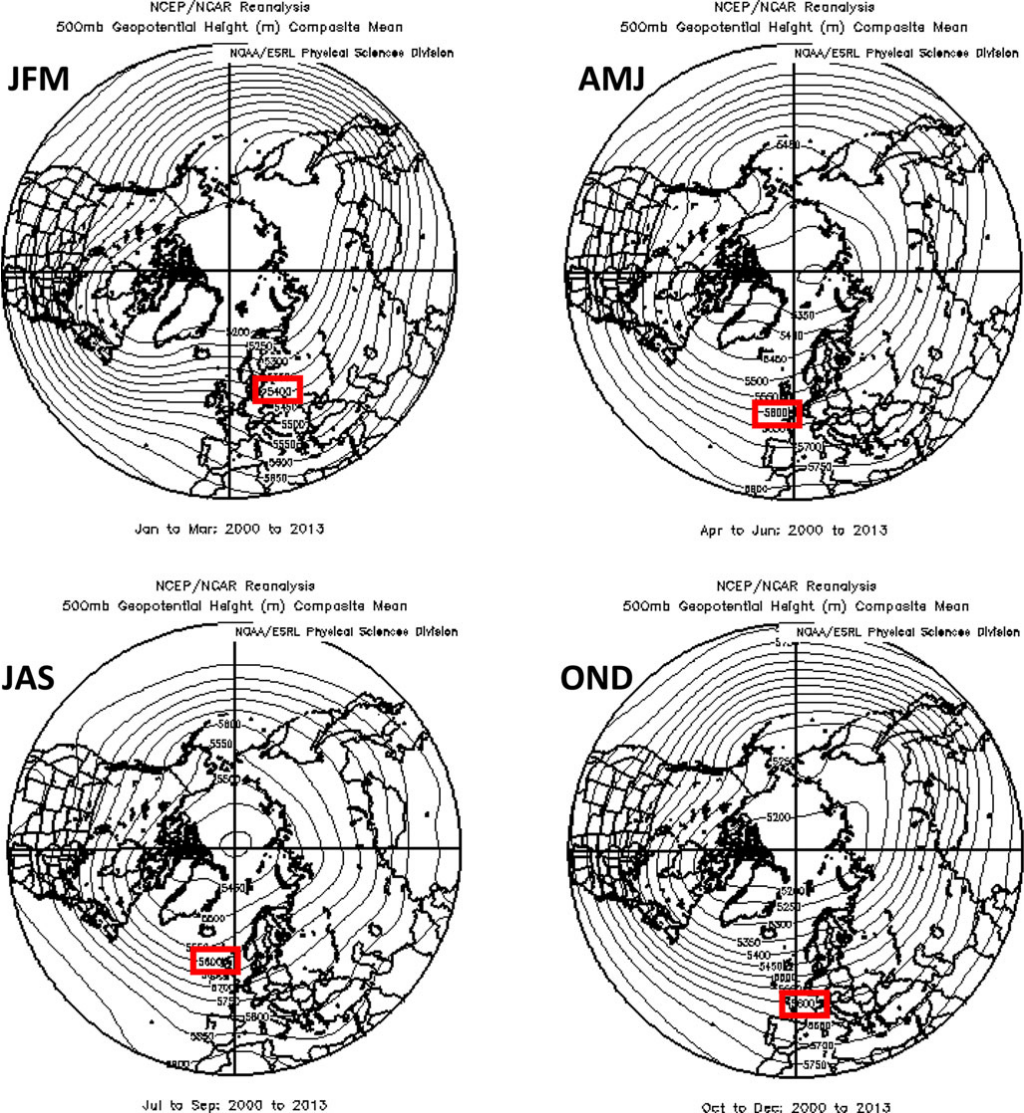
Average seasonal 500 hPa geopotential height contours for the period 2000 to 2013. The contour used for the analysis of high-amplitude patterns for each season is indicated with a red box. Data were obtained from the NCEP Reanalysis, NOAA/ESRL Physical Sciences Division, Boulder, CO, http://www.esrl.noaa.gov/psd/.

The analysis is based on daily fields of 500 hPa geopotential heights obtained from the NCEP/NCAR reanalysis [24]. A particular height contour is selected that exists in the climatological path of the strongest 500 hPa winds, and thus represents the flow trajectory of the jet stream. The 5600 m contour is used in spring through autumn (AMJ, JAS and OND), and 5400 m is used in winter (JFM). The average positions of these contours in the 500 hPa height field for each season are shown in figure 4. An HAP is identified on days when the difference between the daily maximum and minimum latitudes of a single height contour in a particular region exceeds 35°. This threshold is selected to obtain approximately 20 events per season in the whole Northern Hemisphere along with sufficient numbers in smaller regions, but the main conclusions are not sensitive to small variations in the threshold or to using other height contours within 100 m. Events are summed to create seasonal-mean averages, which are presented in table 1. Frequencies of HAPs during the period prior to the emergence of AA (1979–1994) are compared to frequencies during the ‘AA era’ (2000–2013). Statistical significance of differences is assessed by comparing them to the standard deviation of HAPs during randomly selected groups of 14 years from the pre-AA era. Varying the division between these periods by 5 years earlier or later makes no appreciable difference to the conclusions (FV15). Values and cell colour in table 1 indicate percentage differences in six regions and in each season.

**Table 1.** Percentage change in seasonal frequency of high-amplitude-wave patterns (HAPs) from the pre-AA period (randomly chosen groups of 14 years) to the AA-era (2000–2013). HAPs are identified when the difference between the maximum and minimum latitude of the 500 hPa height contour (selected to correspond with mean height of strongest westerly winds) within a specified region exceeds 35° latitude. Asterisks indicate an exceedance of 1 or 2 s.d. in HAPs during 100 random samples of 14 years from 1979 to 1994. Height data were obtained from the NCEP/NCAR reanalysis, NOAA/ESRL Physical Sciences Division, Boulder, CO, http://www.esrl.noaa.gov/psd/. Adapted from [3].

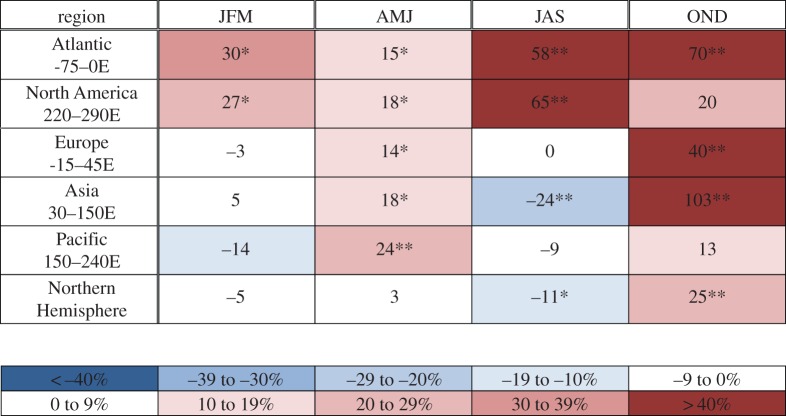

Substantial increases in the occurrence of HAPs have occurred particularly during autumn in most regions, with large increases evident over the Atlantic, Europe and Asia, as well as over the Atlantic and North America during summer. The results for autumn and winter are consistent with the expected response to the large AA in these seasons and support the hypothesis proposed by FV12 and Liu *et al.* [12]. We speculate that increased frequencies in summer may result in part from the rapid decline in late spring snow cover on high-latitude land areas [25]. As snow disappears, bare soil is exposed to the strong spring sunshine earlier, which allows it to dry and warm sooner [25,26]. This effect is at least partly responsible for the approximately 2°C of warming over high-latitude land areas since the mid-1980s [27,28]. Because HAPs have been linked with a variety of extreme weather types [15], our findings suggest that the recent increase in extreme weather events throughout the Northern Hemisphere mid-latitudes [29] may be partly due to the rapid pace of Arctic warming.

## 4. Changes in jet-stream waviness assessed using self-organizing maps

Self-organizing maps (SOMs) are a powerful statistical tool used to extract diagnostic information from large datasets. They provide a means to visualize patterns in the data. The SOMs' neural-network algorithm reduces the dimension of large datasets by grouping similar two-dimensional fields into clusters or ‘nodes’ and organizing them into a matrix of maps with recognizable, intuitive patterns [30–32]. The two-dimensional maps in the SOM matrix provide a more intuitive rendering of pattern characteristics and their relative frequency of occurrence than is achieved by other statistical tools, such as empirical orthogonal functions.

A brief description of the SOM method is presented here; see Skific *et al*. [33] and Skific & Francis [32] for further detail. The initial step creates reference vectors, which represent a first-guess set of characteristic clusters based on the series of individual two-dimensional maps. Each of these vectors has a position or node assigned on a two-dimensional array. The size of the matrix is chosen to balance having enough nodes to capture the important features in the data while being small enough to visually interpret the patterns and display them conveniently. Results are insensitive to small changes in the matrix size [9]. The first-guess reference vectors are derived from the covariance matrix of the two-dimensional fields with the largest eigenvalues. The two largest eigenvectors are placed in the corners of the map, with the central cluster corresponding to the mean of the dataset. The rest of the vectors are derived by using linear interpolation. The vectors are then ‘trained’ by calculating the similarity between each daily field and each of the reference vectors, usually measured as the Euclidean distance in space. The ‘best match’ reference vector and its neighbouring vectors are further refined in an iterative process so that the final set of vectors best approximates clusters of the daily data [30,34]. The resulting clusters become organized on a two-dimensional array such that more similar patterns are placed closer together, while those less similar are farther apart, allowing a more intuitive interpretation of patterns and their relationship to each other in the matrix. Once all the individual daily fields have been assigned to their ‘best match’ cluster, the frequencies of occurrence can be determined, i.e. the fraction of days that reside in each cluster, as well as changes over time in both frequency and cluster-mean values.

In this study, we use SOMs in a novel way. Rather than creating the master SOM with a time series of two-dimensional maps, we instead use a single contour (5600 m to coincide with the climatological height of maximum zonal wind speed) from the daily fields of 500 hPa heights over the Northern Hemisphere. This allows an analysis of the meridional waviness in the large-scale circulation. The mean latitude is first subtracted from each daily contour to remove seasonal latitude migrations and to obtain daily contour anomalies.

**Figure 5.**
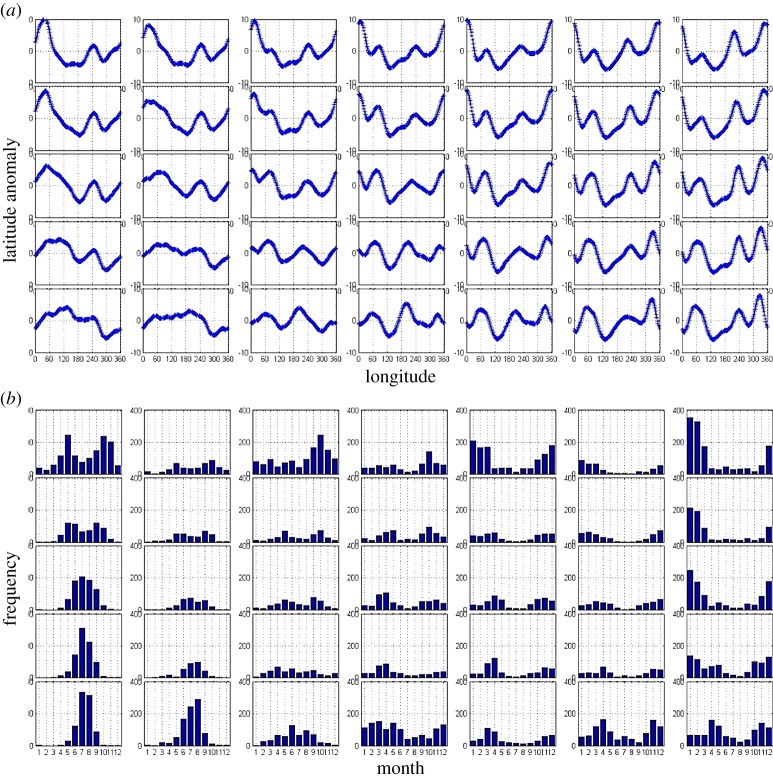
Master SOM of daily 5600-m 500 hPa height contours during 1948 to 2012 (*a*). The daily mean latitude of each contour is subtracted to obtain latitude anomalies. Monthly frequency distribution of days belonging in each SOM cluster (*b*). Data were obtained from the NCEP Reanalysis, NOAA/ESRL Physical Sciences Division, Boulder, CO, http://www.esrl.noaa.gov/psd/.

The master SOM created from this dataset is shown in figure 5*a*. Each pattern in the matrix represents a characteristic configuration of the jet stream's path. One of the advantages of SOM analysis is that many of the patterns are recognizable to a meteorologist, such as the upper-right corner featuring a distinct ridge/trough/ridge system from western North America across the Atlantic, or the upper-left corner with a familiar ridge/trough couplet over Europe and Asia.

**Figure 6.**
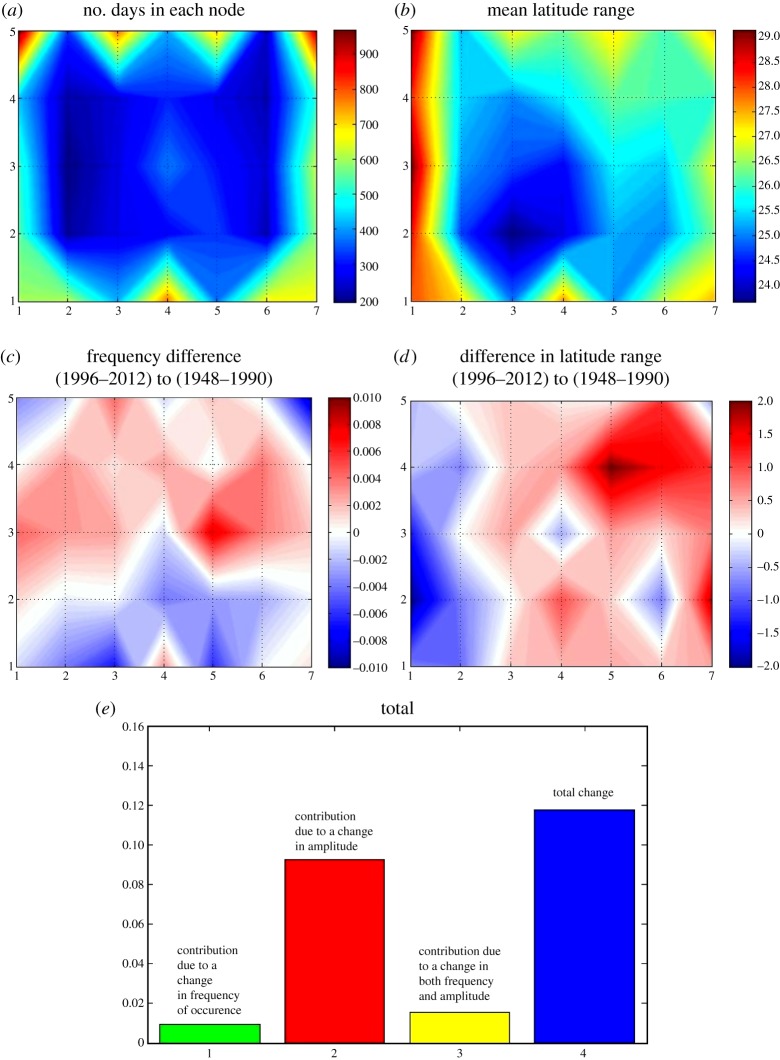
(*a*) Number of days that belong in each node of the master SOM. (*b*) Mean latitude range (deg.) of contours in each node. (*c*) Difference in frequency of days (number of days relative to total days) from earlier to later periods. (*d*) Change in mean latitude range (deg.) from earlier to later period. (*e*) Relative contributions by each factor to annual-mean total change in the SOM-mean latitude range.

Because it is known which days belong in each cluster, a monthly frequency distribution can be constructed, shown in figure 5*b*. From these histograms corresponding to the master SOM, it is apparent that patterns in the upper-right part of the matrix tend to occur in winter, lower-left patterns in summer, and those in the middle of the matrix occur throughout the year but are relatively rare. Figure 6*a* bears this out, as it presents a contour map of the number of days that belong in each cluster. Patterns around the perimeter of the SOM tend to occur most frequently. Using the group of days that belong in each pattern, one can also calculate the mean latitude range for each cluster, which is shown in figure 6*b*. Summer patterns, which tend to occur along the left edge of the matrix, have the highest mean wave amplitudes. The predominantly winter patterns in the upper-right also have high values.

This information can be used to investigate changes in frequency and mean amplitude over time. We calculate the difference between the early part of the record (1948–1990) before the signal of AA emerged and the later period (1996–2012). These differences are shown in figure 6*c*,*d*. An increase in frequency (fraction of days relative to total for each time period) is apparent in a broad region of the matrix from left to right, with decreases generally along the bottom third. More interesting is the change in cluster-mean amplitude: most clusters in the right two-thirds of the SOM exhibit increasing latitude ranges. Largest increases appear for predominantly winter patterns in the upper right.

Using a technique developed by Cassano *et al*. [35], the total change in waviness can be separated into the components related to changing fraction of days in which the atmosphere resides (so-called dynamic factor) and the contribution from a change in the cluster-mean quantity of interest, in this case, amplitude. The dynamic factor is calculated by multiplying the change in frequency of each cluster over time by the amplitude during the early period. The amplitude factor is the product of the change in amplitude and the early-period frequency. A usually small combined term is the product of the time-change in amplitude and time-change in frequency. (For further detail on this technique, refer to [32,35]) The results of this analysis appear in figure 6*e*. The total change in amplitude, averaged over the entire SOM, is represented by the blue bar on the far right, with contributing factors shown to the left. It is clear that the change in cluster-mean amplitude contributes the lion's share to the total change, with positive contributions also from the other factors. When segregated by season (not shown), this relationship is maintained, except in summer when the total change and amplitude factor are both negative, consistent with the decreasing amplitudes for summer patterns apparent in figure 6*d*.

These results are generally consistent with those of the HAPs analysis. Patterns that occur frequently in late autumn (upper-right of SOM matrix) are increasing, especially those with large wave/trough configurations over North America and the Atlantic. Clusters in the upper-middle part of the SOM that feature high-amplitude configurations over the same region are also increasing. Further investigation is ongoing to understand the relationship between these two new metrics.

## 5. Conclusion

Many examples of ‘stuck’ weather patterns during the past few years come to mind. Deep troughs in the jet stream hung over the U.S. east coast and western Europe during the winters of 2009/2010, 2010/2011 and 2012/2013 bringing a seemingly endless string of snow storms and bone-chilling cold. In the early winter of 2011/2012, in contrast, these same areas were under ridges, which brought unusually warm and snowless conditions, while at the same time a deep trough sat over Alaska, producing record snowfalls. During summer, persistent weather patterns have caused severe droughts and heat waves over the Northern Hemisphere's continents. The record high temperatures in Europe and Russia have been linked to early snowmelt in Siberia [36]. When Hurricane Sandy tracked up the eastern seaboard during late October 2012, a high-amplitude trough-ridge pattern was in place over North America and the North Atlantic, creating the flow that steered the storm on its unprecedented westward path into New Jersey. A large ridge in the northwest Atlantic generated a strong surface high-pressure cell that, together with Sandy, formed the steep pressure gradient that caused an expansive area of destructive tropical-storm-force winds from Delaware to Nova Scotia. Sandy occurred after a summer of record-shattering Arctic sea-ice loss, but whether the two phenomena were connected is unclear [37].

While it is difficult to say with any certainty that AA is the cause of any particular extreme weather event, these are the types of phenomena that are expected to occur more often as the world continues to warm and the Arctic continues to lose its ice. Clearly, much additional research is needed to understand better the mechanisms by which mid-latitude weather patterns will respond to anthropogenic climate change, and particularly if and how they may be influenced by AA. There is also much to learn about the interplay among AA and modes of natural variability (such as the El Niño Southern Oscillation, Pacific Decadal Oscillation and Atlantic Multidecadal Oscillation). The recent flooding in the UK (winter 2014) and the North America ‘Snowmageddon’ (February 2010), for example, were apparently caused by a combination of Arctic and tropical influences on the jet stream's configuration [20,21]. Progress can be made by assessing the behaviour and trends in weather patterns by region and season, as the globe—and particularly the Arctic—continue to warm in response to unabated emissions of greenhouse gases.
